# Ready for the Triple Aim? Perspectives on organizational readiness for implementing change from a Danish obstetrics and gynecology department

**DOI:** 10.1186/s12913-019-4319-3

**Published:** 2019-07-24

**Authors:** Marie Höjriis Storkholm, Carl Savage, Mesfin Kassaye Tessma, Jannie Dalby Salvig, Pamela Mazzocato

**Affiliations:** 1 Medical Management Centre, Department of Learning, Informatics, Management and Ethics, Karolinska Institutet, Tomtebodavägen 18A, 171 77 Stockholm, Sweden; 2 Department of Obstetrics and Gynecology, Aarhus University Hospital, Aarhus, Denmark; 3 Medical Statistics Unit, Department of Learning, Informatics, Management and Ethics, Karolinska Institutet, Stockholm, Sweden

**Keywords:** Change leadership, Change management, Health care reform, Organizational readiness for change, Triple aim

## Abstract

**Background:**

As health care strives towards the Triple Aim of improved population health, patient experience, and reduced costs, an organization’s readiness for change may be a key factor. The concept refers to the collective commitment of organizational members to a change and belief in their shared ability to make that change happen (efficacy). This study aims to assess the organizational readiness for implementing large-scale change at a clinical department in pursuit of the Triple Aim and to determine key associated factors.

**Methods:**

A cross-sectional study at a Danish Obstetrics and Gynecology department faced with external pressure to become more efficient without compromising patient outcomes and experience. The Organisational Readiness for Implementing Change (ORIC) questionnaire was distributed to all employees (*n* = 403). Descriptive statistics was used to assess overall organizational readiness and single items. The between-group differences in subject characteristics were assessed with independent t-test and non-parametric test. Multiple linear regression was employed to control for potential confounders.

**Results:**

Response rate was 72%. The level of agreement with the commitment statements was high, and low with the efficacy statements. We did not observe statistically significant differences in the overall score between organizational sections or in relation to gender, age, or profession. Managerial status (B = 3.2, 95% CI = .52, 5.9, *P* = .02) or interim employment(B = 2.7, 95% CI = .47, 4.9, *P* = .02) were significant predictors of a high change efficacy score after controlling for potential confounders.

**Conclusions:**

Changes related to pursuit of the Triple Aim were seen as something that “has to” be done, but left managers, and even more so staff, wondering what “to do” and “how to” do it. Change strategies should therefore address these uncertainties by translating political “have to’s” proposals that resonate with staff, spark engagement, and clarify “how to” deal with the complexity of large-scale change.

## Background

Achieving the Triple Aim [1, 2], i.e. to reduce per capita cost, while improving population health and patient experience, requires health care organizations to undergo considerable change. [2] Downsizing, mergers, or the construction of new hospitals are the most common cost-reduction strategies, yet they are difficult to implement and often fail to deliver the desired results. [3, 4] Moreover, they can have negative effects on employees’ well-being [5–7], and subsequently on patient care. [8]

Successful change is influenced by multiple factors, including perceived differences in mental models, values, and an organization’s readiness for change. [9] Health care professionals can perceive the Triple Aim as inherently paradoxical, and the conflicting mental models of staff and managers can make the Triple Aim challenging to achieve in practice. [10, 11] It has been argued that a change is more successful if it is perceived as congruent with employees’ values. [9] And how employees perceive their organization’s readiness for change (ORC) can influence the success or failure of change initiatives. [9, 12, 13]

Organizational readiness for change is defined as, “the extent to which organizational members are psychologically and behaviorally prepared to implement organizational change”. [12] Two dimensions are essential: “the collective determination of members to implement a change (change commitment)” and “the shared belief in their ability to do so (change efficacy)”. Thus, the focus is on the collective supra-individual level of an organization, rather than at the individual level. Championing behavior (e.g., promoting the value of the change to others) [9, 14] and more cooperative behavior (e.g., volunteering for problem-solving teams) are displayed, if *commitment* to change is based on “want to” rather than “have to” or “ought to”. [9] The level of *efficacy* reflects the perceived knowledge about contextual factors, task demands, and the resources available. [15, 16]

Multiple frameworks and tools to measure this construct in health care exist. However, many present methodological and conceptual limitations. [12] The “Organizational Readiness for Implementing Change” (ORIC) questionnaire has several strengths. It is brief, and thus suitable to be used in busy health care organizations. It is theory-based and psychometrically validated to measure readiness for change at the supra-individual (collective) level. [16] It has been used prior to implementing specific changes, such as the introduction of electronic medical records systems, [17] educational and wellness programs, [18, 19] and quality improvement initiatives. [20–22] However, we have not found that ORIC has been explored in the context of major efficiency requirements in a hospital setting, even though this is a common challenge for health care. A “real world” empirical study of an organization-wide change effort in a clinical department can contribute to the field of readiness for change currently dominated by research on specific or single improvement initiatives or programs. Therefore, this study aims to assess organizational readiness for implementing large-scale change at a clinical department in pursuit of the Triple Aim and to determine associated key factors.

## Methods

This cross-sectional survey study was conducted in the Obstetrics and Gynecology (OB/GYN) department at Aarhus University Hospital (AUH), in Denmark.

### Setting

The Danish health care system is undergoing a major reform that includes large-scale structural changes of hospitals to improve integration, coordination, and capacity utilization. [23] This redesign has included mergers, renovations, and the construction of new hospitals. [24, 25]. Locally in AUH, this has led to a demand on department managers to downsize beds, budget and staff, without compromising health outcomes and patient experience. Thus, this case captures well the challenges of pursuing the Triple Aim, as described in our previous publication. [10]

The specific efficiency requirements set in May 2013 for the OB/GYN department, were to reduce beds by 33% and reduce the budget by 10%, mainly through reductions in nursing staff. The department had ca. 400 employees, 70 beds, 8500 admissions, 100,000 outpatient visits organized in two sections (Gynecology and Obstetrics). The downsizing goals were achieved through significant changes in service delivery pathways, rather than through new organizational structures or layoffs. [10]

Selected staff from different professions met together with clinical managers to revise 46 clinical pathways in a series of off-site workshops using a lean-inspired approach. [26] In interdisciplinary workshops, they focused on reducing “waste” and designing more efficient care pathways. The change strategy was referred to by department managers as the “professional path” and emphasized that the purpose of mapping and revising care processes was to improve, not just to cut costs. An in-depth and exhaustive analysis phase was followed by an iterative process of managerial prioritization, new working groups, and implementation driven by middle managers, selected staff, and department managers.

The clinical pathways for thirty-seven individual conditions and seven multiple conditions were redesigned, and nine changes were made at the departmental level. The latter addressed referrals, physical layouts, flow and capacity, discharge speed, and managerial support. Between 2013 and 2016, overall expenses were reduced by 8.6, 33% of beds were closed, and productivity improved (the number of outpatient visits, admissions and surgeries was stable, while length of stay was reduced). Cost reduction was achieved mainly through reductions in nursing staff, who either voluntarily left for other positions or retired.

### The questionnaire

We used the “Organisational Readiness for Implementing Change” (ORIC) questionnaire. [16] We translated and validated a Danish version of ORIC as reported in a previous publication, which resulted in an 11-item (instead of the original 12) two-factor scale (commitment and efficacy) to be valid. [27] The original efficacy item, “People who work here feel confident that the organization can get people invested in implementing this change” was excluded due to cross loading. The 11-item questionnaire was used for the statistical analysis presented in this study. In addition to the translation, and as recommended by Weiner, [9] the distributed questionnaire was modified through the inclusion of a description of the organizational change in the introduction and the change that was referred to was specified in the relevant item sets. Questions about employment, position, gender, and affiliation to either the gynecological or obstetrical section of the department were added.

To facilitate analysis, we grouped and labelled the items according to the domain they addressed (Table 1). Staff and managers were asked to rate their level of agreement with items measuring efficacy and commitment with a 5-point Likert scale (1 = strongly disagree and 5 = strongly agree). The efficacy-score reflects the organization’s perceived ability to support the change and a low commitment-score indicates resistance to the expected change. [16]

**Table 1:** The original version of the ORIC (12 items) as presented in (Shea et al., 2014)

Item Number	Item Description
Change Efficacy (7 items)	
E1	People who work here feel confident that the organization can get people invested in implementing this change
E2	People who work here feel confident that they can keep track of progress in implementing this change
E3	People who work here feel confident that the organization can support people as they adjust to this change
E4	People who work here feel confident that they can keep the momentum going in implementing this change
E5	People who work here feel confident that they can handle the challenges that might arise in implementing this change
E6	People who work here feel confident that they can coordinate tasks so that implementation goes smoothly
E7	People who work here feel confident that they can manage the politics of implementing this change
Change Commitment (5 items)	
C1	People who work here are committed to implementing this change
C2	People who work here will do whatever it takes to implement this change
C3	People who work here want to implement this change
C4	People who work here are determined to implement this change
C5	People who work here are motivated to implement this change

### Data collection

The questionnaire was administrated electronically via SurveyXact (Aarhus/Denmark) and distributed (as a closed survey) to all department staff and managers (*n* = 403) in June 2014 via email. All managers had a clinical background as either physician, midwife, nurse, or medical secretary. The group of managers included both the department heads and the first line managers. Only managers with direct responsibility for personnel were included.

The survey was conducted as part of larger study that investigated the downsizing initiative from multiple perspectives. The larger study had been presented to the entire department. In addition, all participants were informed about the survey when they received the invitation via e-mail. Distribution was synchronized with the change process to ensure that we captured the organization’s readiness for implementing change at the timepoint when all staff was aware of the considerable changes that were going to be implemented but before actual implementation had begun. The point chosen was after the initial analysis of 46 clinical pathways had been performed. Staff was regularly informed about the change process through e-mail newsletters, plenary meetings, staff meetings and a blog, in the period from the initial planning (April 2013) to implementation. The implementation process began on July 1, 2014, which was when the majority of beds were closed and changes in the care for individual medical conditions and the organization of care units was initiated. However, as this was the start of the summer vacation period, reminders were sent out through September to ensure that all employees received information about the questionnaire. Thus, data collection was conducted from June to September 2014.

Participants were informed of the purpose and length of time of the survey, that data collection, analysis and access to the pseudo-anonymized data-set was limited to the research team and that it was possible to withdraw at any point. Respondents were then asked to give their consent to participate at the commencement of the questionnaire.

### Data analysis

Descriptive statistics was used to analyze the respondents’ characteristics, the overall organizational readiness for implementing change, and the ratings (%) of the single items. Means, standard deviations for continuous variables, medians (interquartile ranges) were used for numerical and ordinal variables, and frequencies or percentages for categorical variables. The between-group differences in subject characteristics were examined using the independent *t-*test for continuous variables. For ordinal variables, the non-parametric Kruskal-Wallis test was used. For evaluating effect size, the difference between means and 95% confidence intervals was used.

Multiple linear regression was employed to control for potential confounders. The total ORIC, efficacy, and commitment scores were the dependent variables. Explanatory variables were selected based on earlier research [27] and simple regression. Categorical explanatory variables were coded depending on their level. If only two (for example, the variable “group” with two categories of staff and manager), the reference category was indicated. Managers were the reference category for the variable “Group”. The associations were presented as regression coefficients (B) with 95% confidence intervals (CI). Residual plots, normal probability plots, and Cook’s distance assessed model assumptions. The final parsimonious model for each outcome variable is presented below. The model building procedure and the guidelines for reporting regression analysis have previously been described in detail elsewhere. [28–30] All statistical analyses were done using the Statistical Package for the Social Sciences (SPSS) for Windows (version 25; IBM, NY, USA) with the level of significance set at 0.05.

Sample size was calculated based on Myers et al. [31] criteria that include: *N* ≥ 200, ratio of N to the number of variables in a model (p), N/*p* ≥ 10. The minimum sample size was satisfied, with a final sample size of 284. Completeness was checked after the questionnaire had been submitted. All items were completed. The full dataset was used with no missing value.

The reporting followed the checklist for Reporting Results of Internet E-Surveys (CHERRIES). [32]

## Results

Response rate was 72%. The frequency distribution of the study population is presented in Table 2. Median length of staff members’ professional experience was 18 years. Median length of employment in the department was 10 years and 1 year for interim staff.

**Table 2:** Frequency distribution of the study population, *n* = 284

Variables	Number (%)
Sex	
Female	264 (93.0)
Male	20 (7.0)
Age group	
18–39	86 (30.3)
40–55	139 (48.9)
56+	59 (20.8)
Section	
Gynecology	85 (29.9)
Obstetrics	162 (57.0)
Both	37 (13.0)
Profession	
Physician	51 (18.0)
Nurse	109 (38.4)
Midwife	95 (33.5)
Secretary	21 (7.4)
Others	8 (2.8)
Work hours	
Part-time	128 (45.1)
Full-time	156 (54.9)
Permanent Employed	
Yes	239 (84.2)
No	45 (15.8)
Interim Staff	
Yes	23 (8.1)
No	261 (91.9)
Manager	
Yes	15 (5.3)
No	269 (94.7)

The overall ORIC score had with a median (IQR, Interquartile range) of 39 (35, 45), with a median change commitment score of 19 (16, 21)and a median change efficacy score of 21 (17, 24).

**Fig. 1:**
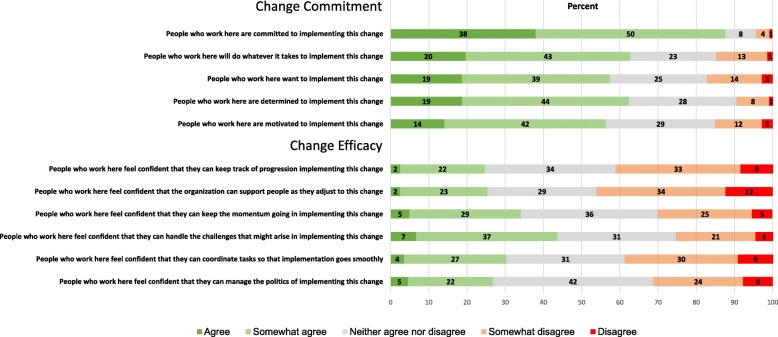
Proportion of response categories by ORIC’s item (*n* = 284)

A majority (56–88%) of the respondents agreed (agree + strongly agree) with the change commitment statements (C1-C5). For change efficacy (E2-E7), this proportion was lower, ranging from 24 to 43% (Fig. 1). 88% of organizational members agreed that the organization was “committed to implementing this change” (C1) and least (56%) with that item that the organization was “motivated to implement change” (C5). Compared to commitment, all efficacy questions were rated lower. Respondents agreed (43%) with “feeling confident that they could handle the challenges that might arise in implementing change” (E6) and least (24%) with “That they could keep track of progress in implementing this change” (E2) (Fig. 1).

We did not observe statistically significant differences in the overall ORIC score between the gynecological and obstetrical sections, neither did gender, age, nor profession influence readiness for implementing change (Table 3). Significantly higher scores in change efficacy and total ORIC were reported by managers and interim staff (this did not include staff in educational positions or those paid hourly wages) (Table 3).

**Table 3:** Mean score (SD) of change commitment, change efficacy, and total ORIC score, *N* = 284

Variables	Commitment	Efficacy	Total ORIC score
	Mean (SD)	Mean (SD)	Mean (SD)
Sex			
Female	18.6 (3.3)	20.9 (5.2)	39.5 (7.7)
Male	18.3 (3.7)	20.5 (5.2)	38.8 (8.1)
Age group			
18–39	19.0 (3.2)	21.1 (4.9)	40.1 (7.2)
40–55	18.3 (3.3)	20.6 (5.1)	38.9 (7.6)
56+	18.9 (3.6)	21.1 (6.0)	40.0 (8.7)
Section			
Gynecology	18.4 (3.3)	20.3 (5.4)	38.7 (7.8)
Obstetrics	18.6 (3.3)	20.9 (5.2)	39.5 (7.7)
Both	19.2 (3.3)	22.1 (5.2)	41.3 (7.6)
Profession			
Physician	18.1 (3.0)	20.9 (4.5)	39.0 (6.5)
Nurse	18.6 (3.6)	20.8 (5.7)	39.4 (8.7)
Midwife	18.8 ((3.2)	20.2 (5.1)	39.0 (7.3)
Medical secretary	19.4 (3.1)	22.4 (4.2)	41.8 (6.5)
Other	19.0 (3.6)	24.0 (4.7)	43.0 (8.1)
Work hours			
Part-time	18.6 (3.6)	20.7 (5.5)	39.3 (8.2)
Full-time	18.7 (3.1)	21.0 (5.0)	39.7 (7.3)
Permanent Employed			
Yes	18.7 (3.3)	20.7 (5.2)	39.3 (7.7)
No	18.6 (3.4)	21.8 (5.2)	40.4 (7.8)
Interim Staff*			
Yes	19.5 (3.9)	23.1 (5.1)	42.6 (8.0)
No	18.6 (3.3)	20.6 (5.2)	39.2 (7.6)
Manager**^†^			
Yes	19.7 (2.3)	23.7 (2.7)	43.4 (4.5)
No	18.6 (3.4)	20.7 (5.3)	39.3 (7.8)

*p < .05 ** *p* < .01 ^†^ = *p*-value for unequal variance reported

Regression analyses were done to control for potential confounders. When controlled for age and gender, the analyses revealed that group (manager vs staff) and interim employment were significant predictors of the dependent variables change efficacy score and total ORIC score (Table 4, Model 1 and Model 3 respectively). For the dependent variable, total efficacy score model 2 showed that “manager” (B = 3.2, 95% CI = .52, 5.9, *P* = .02) and interim employment, yes (B = 2.7, 95% CI = .47, 4.9, *P* = .02); were significant predictors (Table 4). For the dependent variable, total ORIC score of the final parsimonious model revealed that “Manager” (B = 4.4, 95% CI = .44, 8.4, *P* = .03) and interim employment, yes (B = 3.6, 95% CI = .37, 6.9, *P* = .03) were significant predictors (Table 4, Model 4). We did not observe statistically significant relationships for the dependent variable “change in commitment score” (data not shown). We did not observe violations of assumptions of independent *t*-test, regression analysis, multicollinearity or serious outlier problems.

**Table 4:** Results of the multiple regression for the dependent variables change efficacy score and total ORIC score

Variable^a^	B	SE	*t*	*P*-value	95% CI
Change efficacy score: Model 1					
Age	.016	.031	.51	.61	−.05, .08
Gender	.30	1.20	.25	.80	−2.1, 2.6
Manager	3.18	1.38	2.31	.022	.47, 5.9
Interim Staff	2.91	1.22	2.39	.017	.51, 5.3
Change efficacy score: Model 2					
Manager	3.2	1.37	2.3	.019	.52, 5.9
Interim Staff	2.7	1.12	2.4	.018	.47, 4.9
Total ORIC score: Model 3					
Age	.00	.046	.007	.99	−.09, .09
Gender	.61	1.78	.34	.73	−2.9, 4.1
Manager	4.4	2.04	2.2	.03	.42, 8.4
Interim Staff	3.6	1.80	2.0	.045	.09, 7.2
Total ORIC score: Model 4					
Manager	4.44	2.03	2.19	.03	.44, 8.4
Interim Staff	3.64	1.66	2.19	.029	.37, 6.9

*B* Coefficient (B), *SE* Standard Error, *t* –test, *CI* Confidence Interval

^a^In all the models we have included the variables age and gender

## Discussion

With this study, we add to the growing body of research about organizational readiness for change in health care. In the specific case of the clinical department studied, descriptive statistics suggest that the majority of the organizational members were committed (agree or strongly agree) to implement large-scale changes in pursuit of the Triple Aim. The percentage of agreement with the efficacy statements was lower and approximately a third of the organizational members answered that they neither agreed nor disagreed. Clinical managers and interim staff scored significantly higher – they perceived the department to be both more ready for the change and have a higher efficacy, i.e. knowledge about “what to do” and “how to do it”.

The descriptive pattern of high commitment and lower efficacy, suggests that, when faced with large-scale changes brought upon through efficiency and downsizing demands, staff and managers may feel uncertain as to whether and how the organization will be able to successfully implement the requisite changes. Among staff, the uncertainty inherent to this complex change may be linked to their perception that the Triple Aim is inherently paradoxical and does not resonate with their mental models. [10] They may alternatively see it as a desirable “stretch goal”, [33] but feel uncertain, i.e. “do not know how to” arrive at the goal.

The pattern of higher commitment and low efficacy can provide important insights for managers facing complex change processes. In a previous study of lean-based improvement in ambulatory care, the pattern was associated with low engagement levels among physicians and non-physicians and burnout among the latter. [21] Working in a busy and stressful atmosphere seems to be associated with a greater perceived need for change, and less perceived support and efficacy for implementing changes. [22] In the clinical department studied here, managers expected that the focus on improvement and involving staff in redesigning care pathways would motivate staff and positively impact the work environment. [10] Our findings suggest, however, that some hurdles remain regarding staff well-being and engagement. Therefore, it could be of crucial importance that efforts to achieve the primary goals of health care, i.e. the Triple Aim, also address a fourth dimension, to improving the work-life of health care, captured in the Quadruple Aim. [34] Managers faced with continual cost-cutting demands may benefit by conceptualizing these demands in terms of improvement in health outcomes and patient and staff experience.

That clinical managers and interim staff perceived the department to have a higher change efficacy than staff could be related to the managerial role and to a lack of organizational history, respectively. Managers were all directly involved in and responsible for the change processes, thus they may have had a better understanding of the resources available, tasks to be completed, and the overall situation. Interim staff were not directly involved in the change process, did not participate in workshops, nor were they engaged as change agents in the implementation. Their median employment length of 1 year may have spared them memories of previous challenging change initiatives, which can be a barrier when organizations have to stretch to reach a challenging goal. [35] Thus, interim staff, without the organizational history and culture, may have expected a highly specialized clinical department in a university hospital, with a high level of clinical expertise and experience, to be able to effectively implement the necessary changes. For the regular staff then, strategies that address and make use of learnings from the past, may help staff to feel more confident as they adjust to the changes implemented.

The lower agreement with the change commitment items linked to “want to” and “being motivated to implement the change” suggests that change commitment could have been related to staff and managers “having to” implement this change as stipulated by the external efficiency demands. This does not resonate well with those staff members who understand the drivers of change in health care to be related to research, evidence, or technology. [10] This would generate a “have to” rather than a “want to” response and a lower belief in their organization’s ability to succeed. Thus, managers could benefit from reframing political “have to’s” as strategies that resonate with professional ideals in order to spark motivation and engagement. [10]

### Limitations/methodological considerations

The generalizability of our findings is limited by the focus on a single department facing a broadly defined Triple Aim challenge. However, this study was part of a larger longitudinal case study, [10] from which we drew considerable contextual knowledge to better interpret the findings. In terms of participant selection, not all those surveyed were actively involved in developing the specific changes in workshops or as participants in working groups. There could therefore be a relationship to the degree of engagement in the change process or in understanding the workings of the hospital organization as a whole, such as we saw with the managers. The strength of the study was the 72% response rate and the timing of the distribution of the questionnaire. A further exploration into the relationship between the ORIC results and degree of engagement in the change process could contribute to a deeper understanding of the role of engagement in how staff deal with change. It could be suggested that interim staff reported significantly higher efficacy scores, could be due to concern for professional consequences for negative responses. However, if that were the case, commitment scores should also have been higher, which was not the case.

## Conclusion

Striving towards the Triple Aim is a complex change process that involves high levels of uncertainly that can negatively impact an organization’s readiness for implementing change, in particular employee’s belief of the organization’s ability to actually implement the changes. More can be done to address the issue of change efficacy, such as strategies specifically targeted to address and deal with staff well-being, the uncertainty associated with large-scale change efforts, and developing increased clarity about “how to” deal with the complexity of change in health care improvement. Translating political “have to’s” into clear strategies that resonate with staff and spark motivation and engagement in order for staff to “want to” is another challenge that managers and researchers should consider exploring in more detail.

## Data Availability

The dataset collected and analyzed during this study is available from the corresponding author on reasonable request.

## Abbreviations

AUH: Aarhus University Hospital
AUH: Aarhus University Hospital
CFA: Confirmatory factor analysis
CFI: Confirmatory fit index
EFA: Exploratory factor analysis
LPN: Licensed Practical Nurses
OB/GYN: Obstetrics and Gynecology
ORC: Organizational Readiness for Change
ORIC: Organizational Readiness for Implementing Change
RMSEA: Root Mean Square Error of Approximation
SPSS, IBM: Statistical Package for the Social Sciences, International Business Machines Corporation
VIF: Variation Inflation Factor
